# Inosine Improves Neurogenic Detrusor Overactivity following Spinal Cord Injury

**DOI:** 10.1371/journal.pone.0141492

**Published:** 2015-11-03

**Authors:** Yeun Goo Chung, Abhishek Seth, Claire Doyle, Debra Franck, Daniel Kim, Vivian Cristofaro, Larry I. Benowitz, Duong D. Tu, Carlos R. Estrada, Joshua R. Mauney, Maryrose P. Sullivan, Rosalyn M. Adam

**Affiliations:** 1 Urological Diseases Research Center, Boston Children’s Hospital, Boston, Massachusetts, United States of America; 2 Department of Surgery, Harvard Medical School, Boston, Massachusetts, United States of America; 3 Department of Neurosurgery, Boston Children’s Hospital, Boston, Massachusetts, United States of America; 4 Division of Urology, VA Boston Healthcare System, West Roxbury, Massachusetts, United States of America; 5 Department of Surgery, Brigham and Women’s Hospital, Boston, Massachusetts, United States of America; Hertie Institute for Clinical Brain Research, University of Tuebingen., GERMANY

## Abstract

Neurogenic detrusor overactivity and the associated loss of bladder control are among the most challenging complications of spinal cord injury (SCI). Anticholinergic agents are the mainstay for medical treatment of detrusor overactivity. However, their use is limited by significant side effects such that a search for new treatments is warranted. Inosine is a naturally occurring purine nucleoside with neuroprotective, neurotrophic and antioxidant effects that is known to improve motor function in preclinical models of SCI. However, its effect on lower urinary tract function has not been determined. The objectives of this study were to determine the effect of systemic administration of inosine on voiding function following SCI and to delineate potential mechanisms of action. Sprague−Dawley rats underwent complete spinal cord transection, or cord compression by application of an aneurysm clip at T8 for 30 sec. Inosine (225 mg/kg) or vehicle was administered daily via intraperitoneal injection either immediately after injury or after a delay of 8 wk. At the end of treatment, voiding behavior was assessed by cystometry. Levels of synaptophysin (SYP), neurofilament 200 (NF200) and TRPV1 in bladder tissues were measured by immunofluorescence imaging. Inosine administration decreased overactivity in both SCI models, with a significant decrease in the frequency of spontaneous non−voiding contractions during filling, compared to vehicle−treated SCI rats (p<0.05), including under conditions of delayed treatment. Immunofluorescence staining demonstrated increased levels of the pan-neuronal marker SYP and the Adelta fiber marker NF200, but decreased staining for the C-fiber marker, TRPV1 in bladder tissues from inosine-treated rats compared to those from vehicle-treated animals, including after delayed treatment. These findings demonstrate that inosine prevents the development of detrusor overactivity and attenuates existing overactivity following SCI, and may achieve its effects through modulation of sensory neurotransmission.

## Introduction

Spinal cord damage from trauma, congenital defects or other insults, can result in loss of normal urinary tract function. As a result of aberrant neural input, the urinary bladders of such patients commonly display extensive fibroproliferative remodeling, resulting in diminished functional capacity, detrusor overactivity and poor compliance. Furthermore, altered neuronal input to the urinary sphincter can cause a loss of coordination between bladder muscle contraction and opening of the sphincter, a condition termed detrusor sphincter dyssynergia (DSD). This in turn results in pathological remodeling of the bladder and ensuing damage to the upper urinary tract predisposing sufferers of spinal lesions to incontinence, recurrent infections and even renal failure.

Current management of detrusor overactivity in individuals with spinal injury focuses on (i) catheterization to circumvent the dyssynergic sphincter and promote bladder emptying; and (ii) medication to reduce involuntary bladder contractions and improve compliance. Existing pharmacotherapy relies heavily on the use of anti-cholinergic agents, which act either through inhibition of neurotransmitter release or by direct receptor blockade (reviewed in [1]). Although medications targeting cholinergic signaling can be effective, they are not without adverse effects. Thus, investigation of alternative strategies to target neurogenic detrusor overactivity is warranted.

Inosine is a naturally occurring purine nucleoside that is generated intracellularly by deamination of adenosine or through the action of 5’-nucleotidase on inosine monophosphate. It possesses antioxidant, anti-inflammatory, axogenic and neurotrophic properties. Inosine is metabolized to hypoxanthine and uric acid, the latter of which is a scavenger of peroxynitrite that mediates antioxidant activity (reviewed in [2]). The neuroprotective activity of inosine has been demonstrated in a range of neuronal cell types in response to a variety of neurologic insults both in vitro and in vivo [3,4,5,6,7,8,9,10,11]. In particular, previous studies have shown that, following unilateral stroke or traumatic brain injury, inosine augments the sprouting of corticospinal tract (CST) fibers from the intact side of the brain to the denervated side of the spinal cord, causing substantial improvements in skilled use of the impaired limbs [4,5,12,13,14]. In these studies, however, the impact of inosine administration on detrusor overactivity following neurologic injury was not explored.

In this study, we investigated the impact of systemic inosine administration on bladder function in transection and compression models of spinal cord injury. We observed significant improvements in detrusor overactivity in SCI rats receiving inosine either immediately at the time of injury or following a delay of 8 wk, compared to vehicle-treated SCI rats. Functional improvements were accompanied by increased immunoreactivity for the pan-neuronal marker synaptophysin and neurofilament 200, and decreased immunoreactivity for TRPV1. These findings suggest inosine acts through modulation of sensory neurotransmission and may be a novel treatment for neurogenic detrusor overactivity.

## Materials and Methods

### Ethics Statement

These studies were performed in strict accordance with the recommendations in the Guide for the Care and Use of Laboratory Animals of the National Institutes of Health. All experiments were approved by the Animal Care and Use Committee of Boston Children’s Hospital (protocol #13-09-2501R). All surgeries were performed under isoflurane anesthesia and every effort was made to minimize suffering.

### Creation of spinal cord injury and inosine administration

Male Sprague-Dawley rats (6–7 wk of age, ~250 g, Charles River Laboratories, Wilmington, MA) were subjected to complete spinal cord transection or spinal cord compression essentially as described [15]. Briefly, under isoflurane anesthesia a dorsal midline incision was made over the thoracic spinal cord. Superficial and deep muscle layers were incised in the midline to expose the spine. For complete transection, the dura was incised, the cord was severed at T8 with a scalpel blade and Gelfoam (Ethicon^™^) was placed between the two cut ends of the spinal cord prior to skin closure; the dura was not closed. For compression, an aneurysm clip imparting 60 g of closing force was applied to the cord at T8 for 30 sec; in this case the dura remained intact. Following closure of the skin incision, post-operative pain was managed with meloxicam analgesia (5 mg/ml, s.c. every 24 h for 3 d). Rats also received prophylactic Baytril (100 mg/ml at 7.5mg/kg, s.c.) during the post-operative period. During the period characterized by bladder areflexia, bladders were emptied every 12 h by manual expression until reflex voiding returned. This period lasted for up to 2 wk in rats subjected to spinal cord transection, and for 5–7 d for rats with cord compression. Rats were administered either inosine (225 mg/kg, i.p.) or a corresponding volume of vehicle as control immediately or following a delay of 8 wk (compression group only). Inosine or vehicle was administered daily for 6 wk. At 6 wk (transection and compression with immediate treatment groups) or 14 wk (compression + delayed treatment group only) after injury, cohorts of rats were subjected to cystometry, bladder contractility testing or tissue harvest for immunohistochemical assessments as outlined below.

### Cystometric analysis

Voiding function was assessed in a subset of injured rats (transection + vehicle, n = 5; transection + inosine, n = 4; compression + vehicle, n = 6; compression + inosine, n = 5; compression + vehicle delayed, n = 6; compression + inosine delayed, n = 3) using conscious cystometry, essentially as described [15]. Cystometry was performed at 6 wk (transection and compression with immediate inosine treatment) or 14 wk (compression with delayed inosine treatment) after creation of injury. Briefly, 3 d prior to analysis, a suprapubic catheter was inserted in a subset of animals in each cohort. Under isoflurane anesthesia, a dorsal midline incision was made in between the scapulae of rats. A laparotomy was created using a lower abdominal midline incision. PE-50 tubing (Intramedic, Sparks, MD) with a flared tip was tunneled from the dorsal incision into the peritoneal cavity, and secured in the dome of the bladder using a purse-string stitch (6–0 prolene). The exteriorized PE-50 tubing on the dorsal aspect was attached to a Luer lock adapter and secured to the skin with a 3–0 silk suture. Post-operative pain was managed with meloxicam analgesia (5 mg/ml, s.c. every 24 h for 3 d), animals also received prophylactic Baytril (100 mg/ml at 7.5mg/kg, s.c.) during the 3 d post-operative period. Conscious cystometry was conducted 3 d after placement of the catheter, by attaching the catheter to a physiological pressure transducer (MLT844, ADInstruments, Colorado Springs, CO) to allow measurement of intravesical pressure, while bladder was continuously infused with sterile PBS at 100 μl/min. After an equilibration period of 60 min, bladder pressures were monitored over 4–5 voiding cycles. Voided volumes were determined by collection of voided bladder contents on a weighing scale placed below the housing cage. Pressure readings were converted to digital signals using a PowerLab data acquisition system and analyzed using LabChart Pro software. Post void residual volumes were measured by aspirating the SP catheter at the conclusion of cystometry. In this analysis, a spontaneous non-voiding contraction (SNVC) was defined as any rise in intravesical pressure of greater than 5 cm H_2_O that did not result in a void. Data from 4–5 voiding cycles were averaged from each animal and included frequency and amplitude of non-voiding contractions, compliance (change in infused volume/change in intravesical pressure during the filling phase), peak pressure during voiding contraction, and voided volumes. Bladder activity was monitored over a period of up to 4 h. At the end of analysis, rats were euthanized via CO_2_ inhalation.

### Ex vivo contractility studies

At the end of the 6 wk treatment period, bladders from a cohort of animals subjected to complete transection and treated with vehicle or inosine (n = 13 each) were harvested and preserved in ice-cold Kreb’s buffer (NaCl 120 mM; KCl 5.9 mM; NaHCO_3_ 25 mM; Na_2_H_2_PO_4_ 1.2 mM; MgCl • 6H_2_O 1.2 mM; CaCl_2_ 2.5 mM; dextrose 11.5 mM) for *ex vivo* contractility analyses. Following euthanasia via CO_2_ inhalation, bladder tissue was carefully cut into strips and the mucosa dissected off the detrusor muscle under microscopic guidance. Detrusor strips were attached to a force transducer (Grass Instruments) and suspended in an organ bath maintained at 37°C and bubbled with a mixture of 95% O_2_ and 5% CO_2_. Following an equilibration period for 45 min, contractile responses to phenylephrine (adrenergic agonist, 100 μM), α,β-methylene-ATP (purinergic agonist, 10 μM), carbachol (1 nM-10 μM), KCl (120 mM), and to electrical field stimulation (1–64 Hz, 20 V, 0.5 ms pulse width, 10 sec duration) were measured in separate strips. Data were expressed as force (mN) normalized by tissue cross-sectional area and presented as mean ± SEM.

### Immunohistochemistry and histomorphometric analysis

Following scheduled euthanasia by CO_2_ inhalation at 6 or 14 wk after injury, bladders were excised for standard histological processing. Briefly, bladders were weighed and fixed in 10% neutral-buffered formalin, and processed for embedding in paraffin. Sections (5 μm) were cut and subjected to immunohistochemical (IHC) analyses using standard procedures, as described [16]. Primary antibodies used were: anti-synaptophysin (SYP38, 1:50 dilution, Abcam, Cambridge, MA], anti-neurofilament (NF200, 1:250 dilution, Sigma-Aldrich, St. Louis, MO), anti-vesicular acetylcholine transferase (VAChT, 1:200 dilution, Santa Cruz Biotechnology, Santa Cruz, CA) and anti-TRPV1 (1:200 dilution, Alomone Laboratories, Jerusalem, Israel). Sections were then incubated with species-matched Alexa Fluor-conjugated secondary antibodies (Life Technologies, Grand Island, NY) and nuclei were counterstained with 4’, 6-diamidino-2-phenyllindole (DAPI). Specimens were visualized using an Axioplan-2 microscope (Carl Zeiss MicroImaging, Thornwood, NY) and representative images were acquired using Axiovision software (version 4.8). Histomorphometric analyses (n = 3–7 animals per group) were performed as previously described [16] to assess the effect of injury and inosine treatment on marker expression using ImageJ software (version 1.47). Area measurements and signal counts were carried out on 8 random, independent microscopic fields (magnification 20X) captured throughout the bladder wall of each animal per injury group (for both vehicle and inosine treatments), for a total of 24–56 independent microscopic fields per group (n = 3–7 animals). These measurements were then summed and averaged across all replicates (non-surgical control n = 4; transection + vehicle n = 4; transection + inosine n = 4; compression + vehicle n = 6; compression + inosine n = 7; compression + vehicle delayed n = 6; compression + inosine delayed n = 3) in each group to determine the extent of SYP, NF200, VAChT or TRPV1 staining. Measurements were normalized to the total tissue area examined (mm^2^). Histomorphometric data were quantified by two independent reviewers to ensure consistency in evaluation, with one observer blinded to the treatment group.

### Statistical analysis

Quantitative measurements for urodynamic and histomorphometric parameters were analyzed as follows: inter-group comparisons between three or more groups were performed with the Kruskal-Wallis test in combination with the Scheffe’s test for post-hoc analysis. Comparison between two groups was performed with Mann-Whitney test. Statistical evaluations were performed with SPSS Statistics software v19.0 (http://www.spss.com) and data were expressed as mean ± standard deviation. Statistically significant values were defined as p<0.05. For contractility testing, differences in contractile responses between treatment groups were determined by Student’s t-test, with p<0.05 considered significant.

## Results

**Fig 1:**
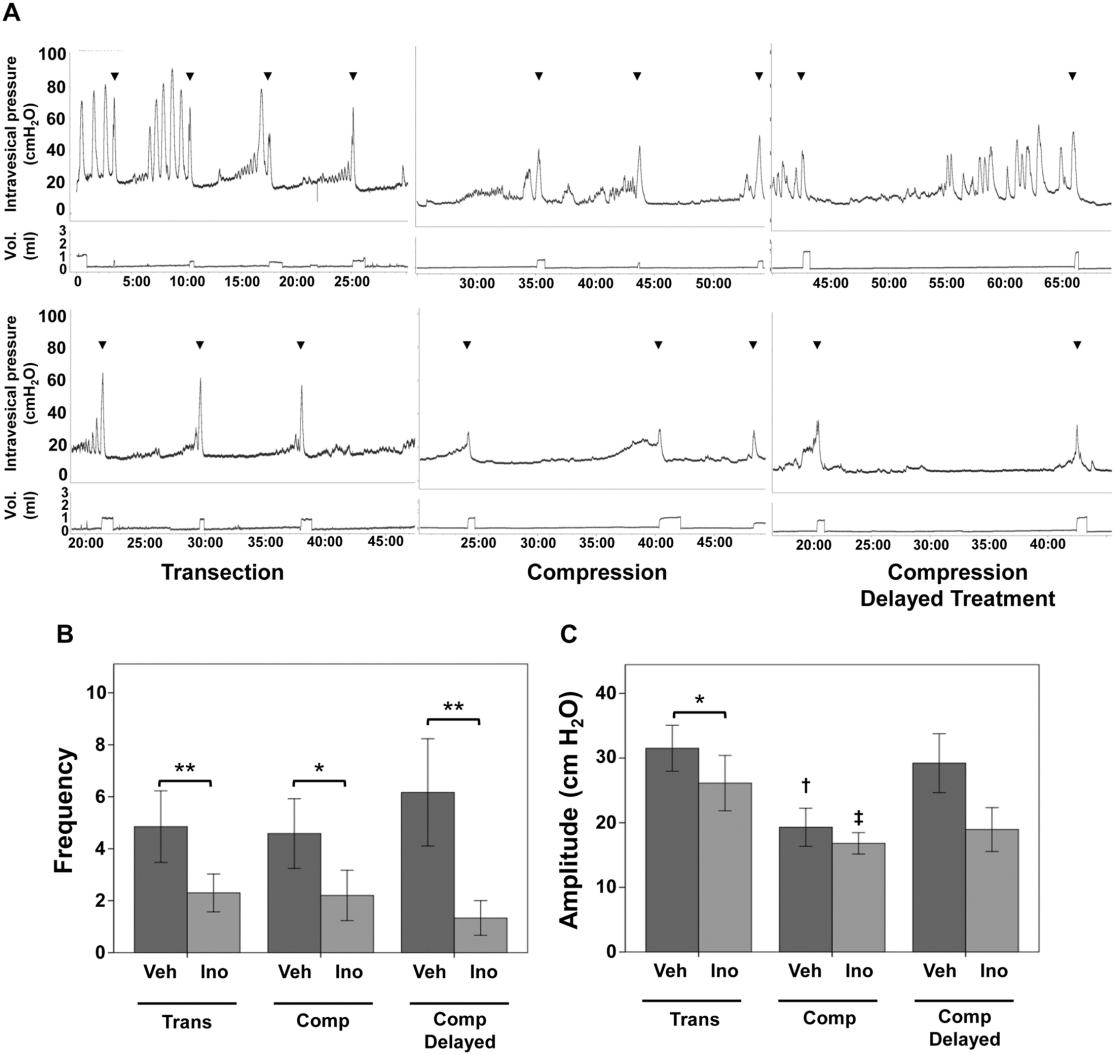
Inosine improves neurogenic detrusor overactivity. **(A)** Representative cystometrograms of rats with transection or compression spinal cord injury that received vehicle (upper panels) or inosine (225 mg/kg, lower panels) i.p. daily for 6 wk, either immediately at the time of injury (left and middle panels) or beginning 8 wk after injury (right panels) are shown. Cystometry was performed at 6 wk (transection and compression with immediate inosine treatment) or 14 wk (compression with delayed inosine treatment). In each case, the upper tracing in each graph demonstrates intravesical pressure (cm H_2_O) and the lower tracing indicates voided volume. Arrowheads indicate voids. Time (in min) is indicated on the x-axis. Numbers of rats in each group are: transection + vehicle, n = 5; transection + inosine, n = 4; compression + vehicle, n = 6; compression + inosine, n = 5; compression + vehicle delayed, n = 6; compression + inosine delayed, n = 3. Graphs summarizing the frequency **(B)** and amplitude **(C)** of spontaneous non-voiding contractions in vehicle- versus inosine-treated SCI rats in both injury models are indicated. Data are presented as mean ± standard deviation. Inosine treatment decreased the frequency of SNVCs in all models, and the amplitude of SNVC following transection injury.

To address the ability of inosine to mitigate detrusor overactivity, one of the primary clinical concerns in patients with SCI, we subjected rats with spinal cord transection or compression injury, to sustained inosine administration, with inosine given for 6 wk either immediately at the time of injury or following a delay of 8 wk. Voiding function was assessed by conscious cystometry performed at 6 wk (transection and compression with immediate inosine treatment) or 14 wk (compression with delayed inosine treatment) after creation of injury. As shown in Fig 1A (upper panels), rats receiving vehicle in all injury groups displayed extensive detrusor overactivity, characterized by frequent spontaneous non-voiding contractions (SNVCs) during conscious cystometry. In contrast, injured rats receiving inosine showed significant attenuation of detrusor overactivity (Fig 1A, lower panels). No significant differences were observed in peak pressure, resting pressure, micturition cycle time, voiding efficiency or compliance (data not shown). Quantification of overactivity revealed reductions in both frequency and amplitude of SNVCs, with >4 times fewer SNVCs compared to vehicle-treated controls (p<0.05)(Fig 1B). Inosine-treated rats in the transection group also showed lower amplitude of SNVCs (p<0.05)(Fig 1C).

**Fig 2:**
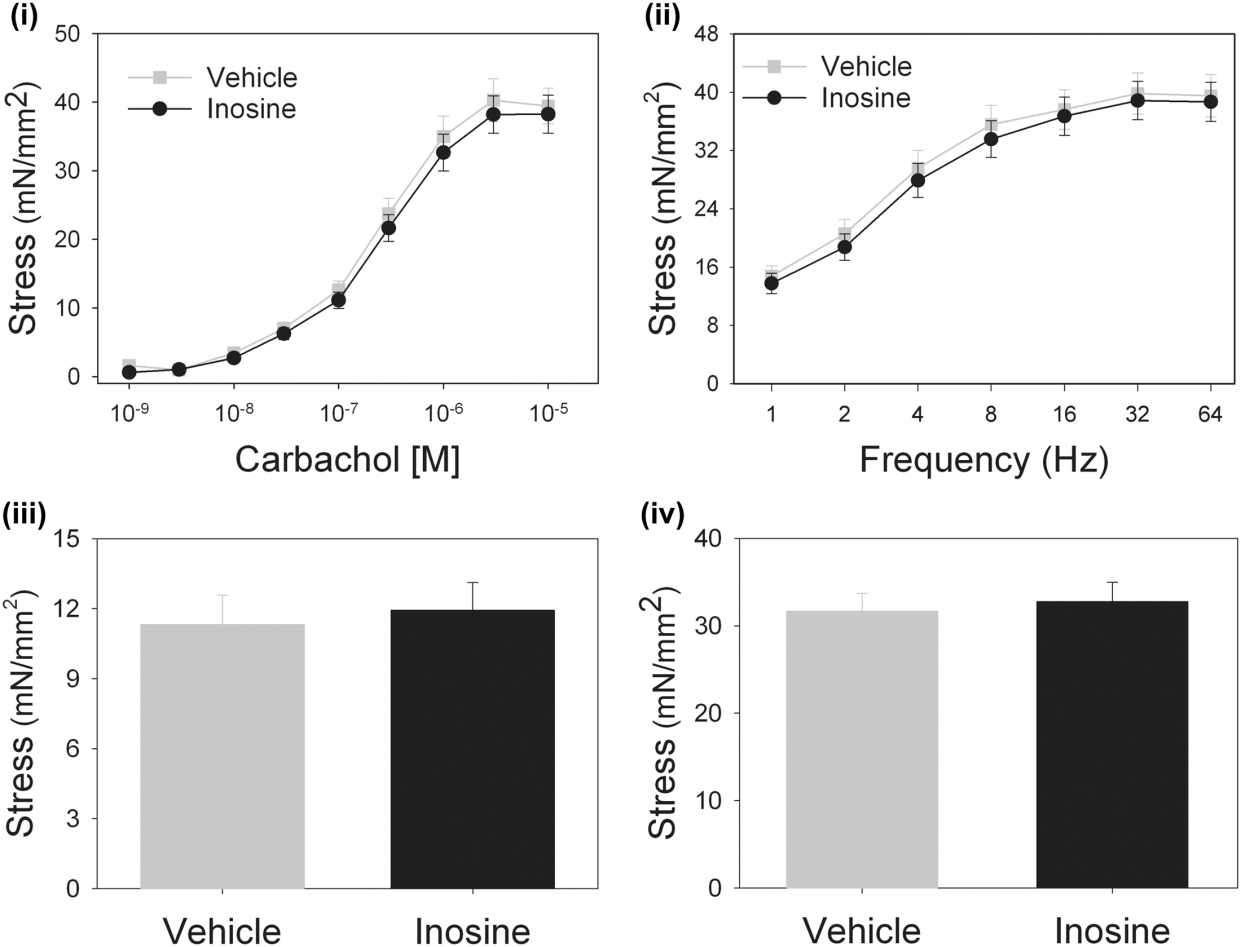
Inosine acts independently of direct effects on smooth muscle contractility. Contractility of muscle strips from vehicle- or inosine-treated rats with transection injury (n = 13 in each group) was assessed in response to (i) carbachol, (ii) electrical field stimulation, (iii) α,β-meATP or (iv) KCl (120 mM). Force generation normalized to cross-sectional tissue area did not differ between tissues from vehicle- and inosine-treated rats under any condition. Data are present as mean ± SEM.

To determine whether the observed improvement in voiding parameters in inosine-treated rats reflected direct effects on muscle contractility, we evaluated agonist-evoked force generation in bladder muscle strips from vehicle- and inosine-treated rats. Muscle contractility induced by phenylephrine, electrical field stimulation, ATP or KCl-mediated depolarization was not significantly different between tissues from inosine-treated or control rats (Fig 2). In addition, immunoreactivity for the vesicular acetylcholine transporter in bladder sections was not significantly different between vehicle- or inosine-treated rats in any of the models, suggesting that the beneficial effect of inosine on voiding function occurred independently of direct effects on muscle contractility.

**Fig 3:**
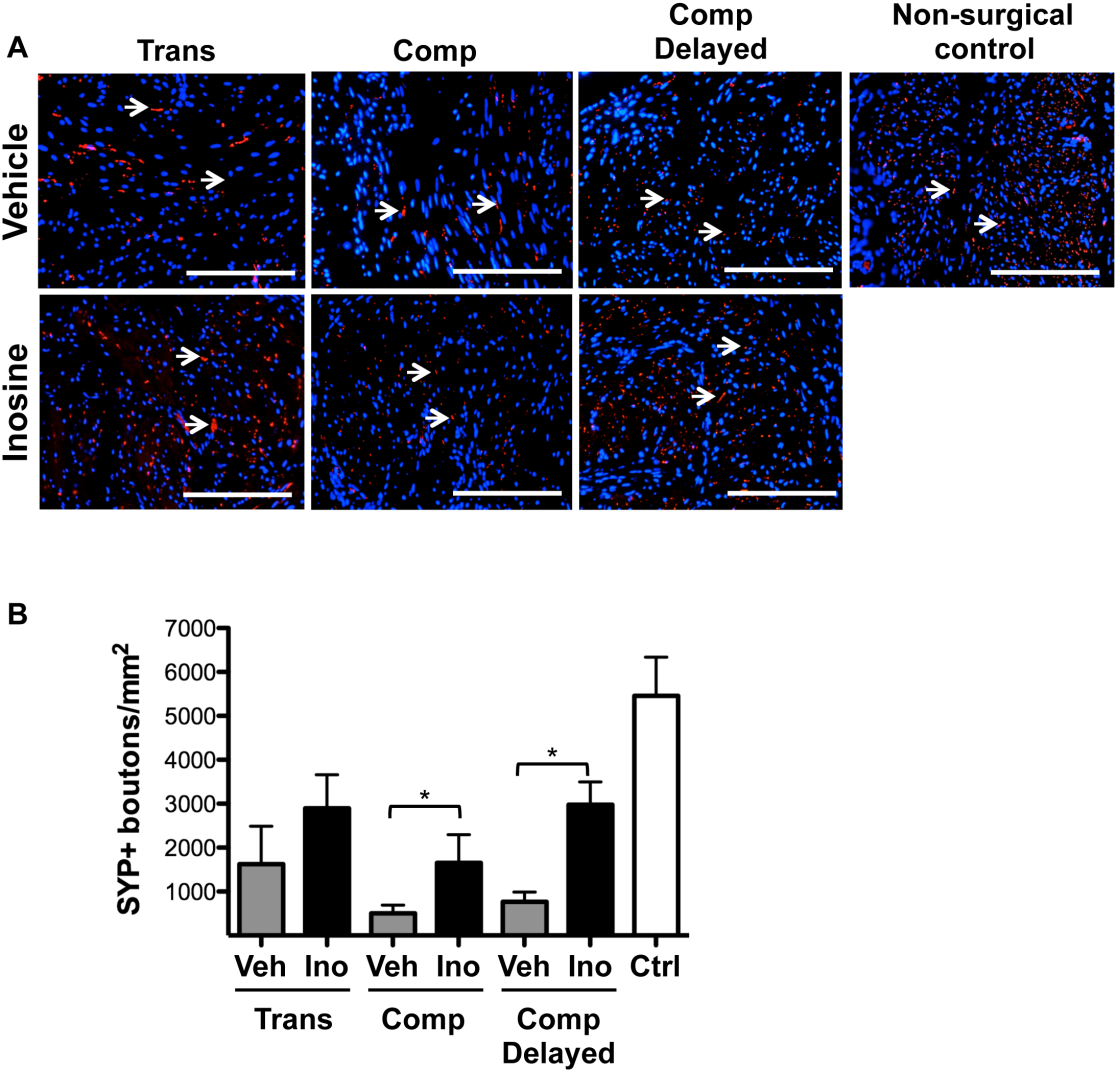
Inosine treatment increases the extent of synaptophysin immunoreactivity following spinal cord injury. (**A**) Representative photomicrographs of staining for the pan-neuronal marker synaptophysin (SYP) in bladder sections from SCI rats treated with vehicle or inosine or non-surgical controls are shown. Numbers of rats in each group were: transection + vehicle, n = 4; transection + inosine, n = 4; compression + vehicle, n = 6; compression + inosine, n = 7; compression + vehicle delayed, n = 6; compression + inosine delayed, n = 3; non-surgical controls (n = 4). For all panels, marker expression is displayed in red (Alexa-Fluor 594) labeling. Blue denotes DAPI nuclear counterstain. Arrows denote SYP+ boutons. Scale bars in all panels = 200 μm. **(B)** Histomorphometric analysis of the density of SYP+ boutons in 8 independent high-power fields. Data are presented as mean ± standard deviation. *, p<0.05 inosine versus respective vehicle control.

Based on our observation that chronic treatment of SCI rats with inosine did not alter motor activity in smooth muscle, and in light of prior studies showing the effects of inosine on axon sprouting [4,6,13,17], we proceeded to assess the impact of inosine treatment on levels of neuronal markers in the bladder. Staining for the pan-neuronal marker synaptophysin (SYP) was decreased in bladder tissues from rats with SCI compared to non-surgical controls. In all injury models, SYP immunoreactivity was increased in bladders following inosine treatment, compared to that observed in tissues from vehicle-treated controls reaching significance in compression injury groups (Fig 3, p<0.05), although signal did not reach the level observed in tissue from non-surgical controls. Importantly, a significant increase in SYP staining was observed even following an 8 wk delay in inosine administration.

**Fig 4:**
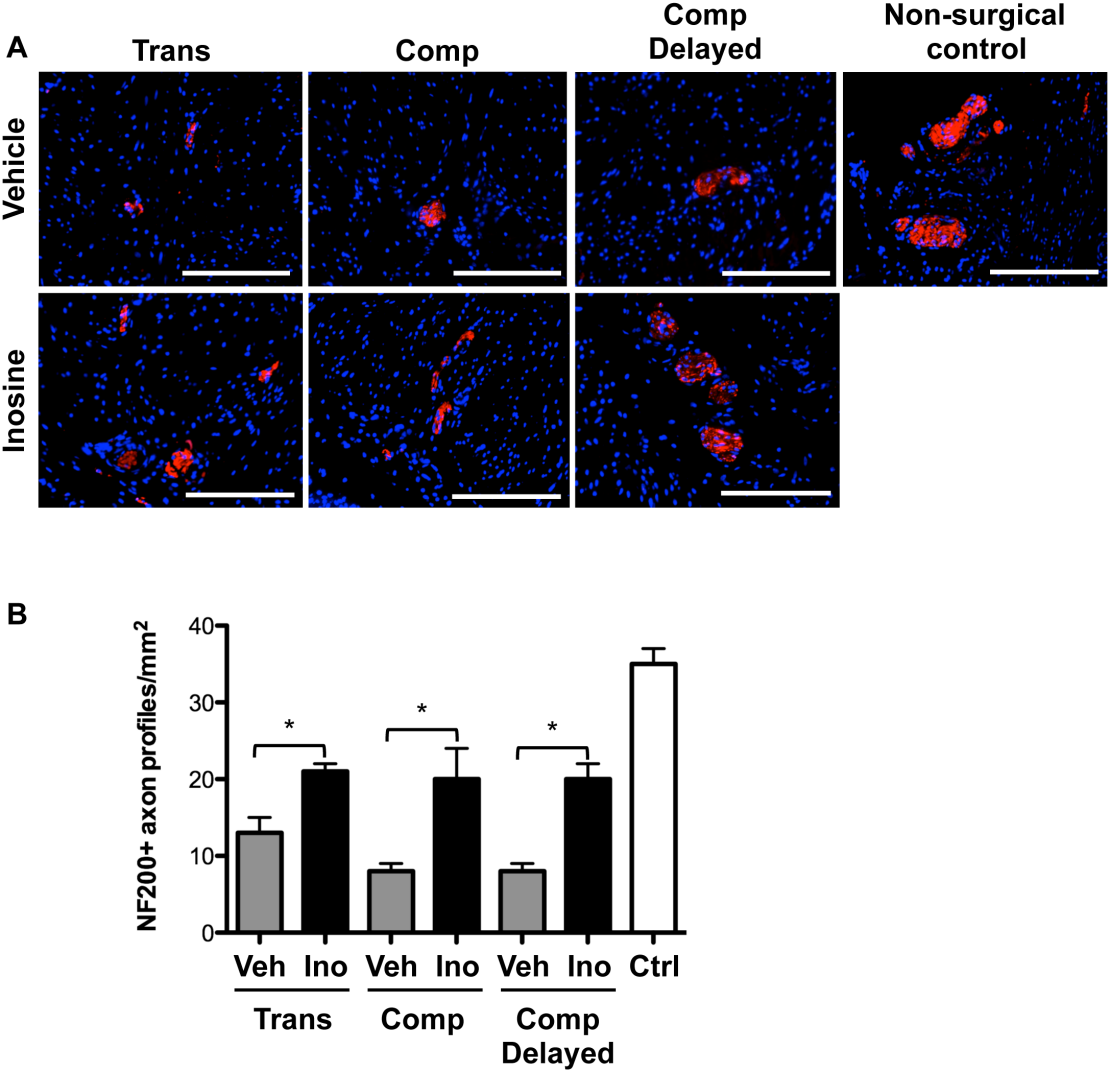
Inosine treatment increases the extent of NF200 immunoreactivity following spinal cord injury. (**A**) Representative photomicrographs of staining for the Aδ fiber marker NF200 (red) in bladder sections from SCI rats treated with vehicle or inosine or non-surgical controls are shown. Numbers of rats in each group were: transection + vehicle, n = 4; transection + inosine, n = 4; compression + vehicle, n = 6; compression + inosine, n = 7; compression + vehicle delayed, n = 6; compression + inosine delayed, n = 3; non-surgical controls (n = 4). Blue denotes DAPI nuclear counterstain. Scale bars = 200 μm. **(B)** Histomorphometric analysis of the density of NF200+ nerve trunks in 8 independent high-power fields. Data are presented as mean ± standard deviation. *, p<0.05 inosine versus corresponding vehicle control.

The number of NF200-positive axon profiles was decreased in bladder tissues from vehicle-treated rats with SCI compared to non-surgical controls. Inosine treatment led to an increase in NF200-positive axon profiles in bladder tissue from all injury groups compared to vehicle-treated animals (Fig 4, p<0.05, all groups). In no case did the number of NF200-positive axon profiles in tissues from inosine-treated rats reach that in tissues from non-surgical controls.

**Fig 5:**
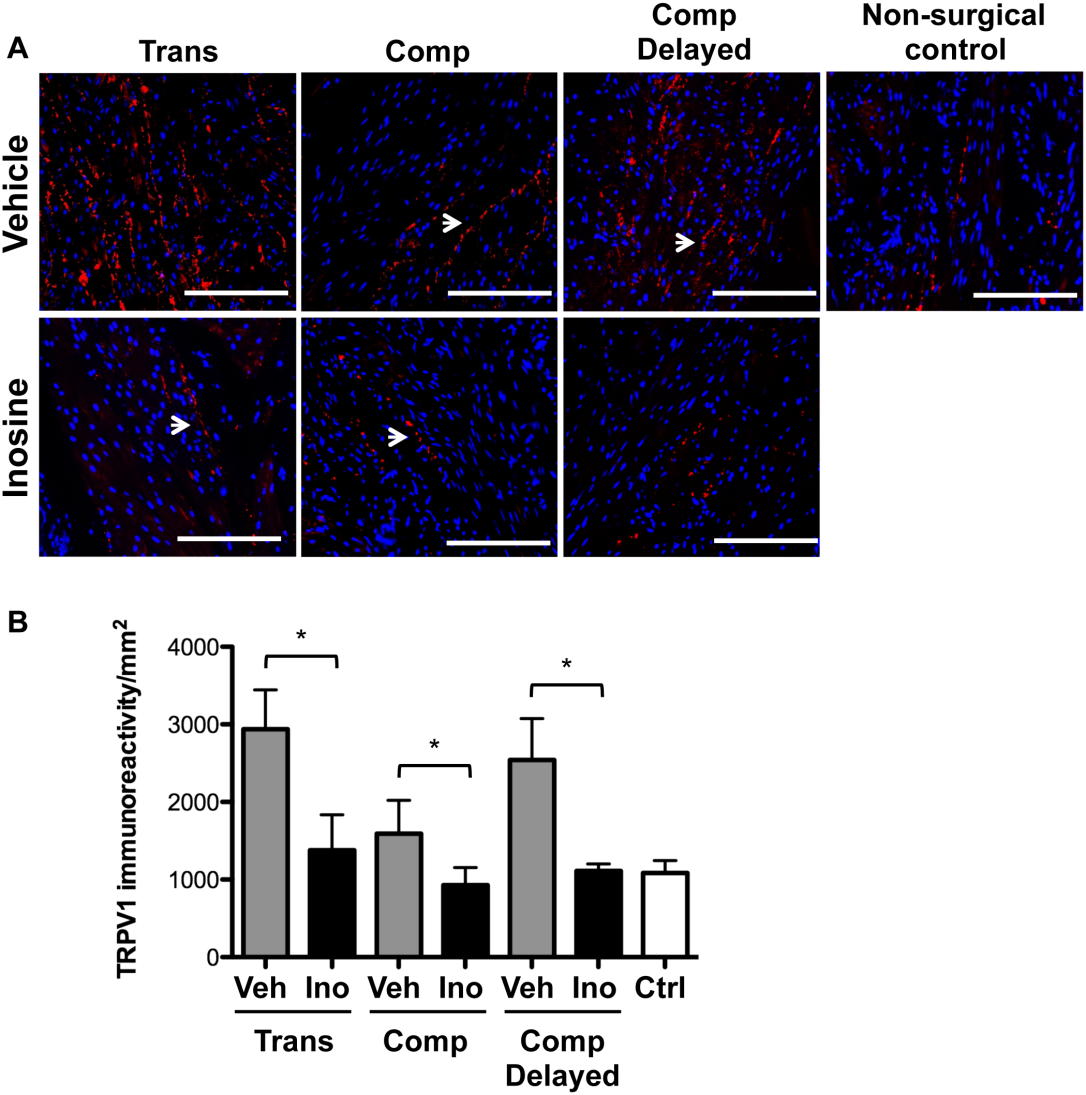
Inosine treatment decreases TRPV1 immunoreactivity following spinal cord injury. Representative photomicrographs of staining for the C fiber marker TRPV1 (red) in bladders from SCI rats treated with vehicle or inosine or non-surgical controls are shown. Numbers of rats in each group were: transection + vehicle, n = 4; transection + inosine, n = 4; compression + vehicle, n = 6; compression + inosine, n = 7; compression + vehicle delayed, n = 6; compression + inosine delayed, n = 3; non-surgical controls (n = 4). Blue denotes DAPI nuclear counterstain. Scale bars = 200 μm. **(B)** Histomorphometric analysis of TRPV1 immunoreactivity in 8 independent high-power fields. Data are presented as mean ± standard deviation. *, p<0.05 inosine versus corresponding vehicle control.

Staining for the C-fiber marker TRPV1 revealed a significant increase in TRPV1 immunoreactivity in the bladders of vehicle-treated rats exposed to each injury condition compared to non-surgical controls (Fig 5, p<0.05). In each condition, inosine treatment was associated with a significant attenuation of TRPV1 immunoreactivity (p<0.05, all groups).

## Discussion

In this study we determined the impact of inosine treatment on lower urinary tract complications following spinal cord injury. Our findings demonstrated (i) a profound improvement in detrusor overactivity in SCI rats receiving inosine compared to vehicle-treated controls in all injury models; (ii) a decrease in the frequency of SNVC in all injury models and a decrease in the amplitude of SNVC in the transection injury group following inosine treatment; (iii) no effect of inosine on motor activity in detrusor smooth muscle; and (iv) increased expression of the pan-neuronal marker SYP and Aδ fiber marker NF200, and decreased expression of the C-fiber marker TRPV1 in bladders of inosine-treated SCI rats compared to vehicle-treated controls. Importantly, several endpoints were improved even in rats receiving delayed inosine treatment. Together, these findings suggest that inosine improves detrusor overactivity in part through modulation of sensory neurotransmission, and that inosine can elicit functional improvements even in the context of existing damage following SCI.

The mechanisms whereby inosine acts to improve neurogenic detrusor overactivity have not been explored. In agreement with prior reports, both transection and compression injury to the spinal cord led to changes in immunoreactivity for the Aδ fiber marker NF200 and the C-fiber marker TRPV1 (reviewed in [18]) in bladder tissues. Previous studies of the impact of spinal cord injury on lower urinary tract function have identified an important role for C-fiber afferent neurons in mediating pathologic changes in voiding behavior [19]. In rodents with detrusor overactivity secondary to SCI or cyclophosphamide-induced inflammation, treatment with the TRPV1 agonists and C-fiber-selective neurotoxins capsaicin or resiniferatoxin was found to inhibit non-voiding contractions through TRPV1 desensitization [20,21]. Our results indicate that inosine treatment attenuated the injury-induced increase in TRPV1 immunoreactivity, both with immediate and delayed administration, in parallel with significant improvements in detrusor overactivity. Together, these findings suggest that inosine achieves its effects, at least in part, through inhibition of TRPV1 expression and/or activity.

The increased SYP immunoreactivity observed in bladders from rats with compression injury that received inosine either immediately or after an 8 wk delay is in agreement with the known neuroprotective and neurotrophic activities of inosine [3,4,5,6,12,13]. In studies investigating regrowth of injured neurons in the central nervous system, inosine was found to promote the growth of axons via activation of the neuron-specific kinase, Mst3b [3,4,5,22,23]. Preliminary observations suggest expression of both Mst3b and Mst3 isoforms in bladder tissue from spinal cord injured rats (R.M.A., unpublished observations); however, the functional significance of Mst3/3b with respect to the potential neurotrophic activity of inosine in the bladder remains to be determined.

Inosine is also known to act through interaction with members of the adenosine receptor family [24,25,26] For example, the anti-nociceptive effects of inosine in diverse models of pain were found to be mediated via A_1_ and A_2A_ subtypes, resulting in inhibition of protein kinase C [25] and protein kinase A, as well as modulation of K_ATP_, K_v_ and BK channels [26]. A_2A_ receptor activation was also implicated in relaxation of bladder muscle strips via K_ATP_ activation and elevation of intracellular cAMP [27], as well as in the immunosuppressive activity of inosine in experimental models of inflammation [24]. In our study, no significant difference in inflammation, as determined by myeloperoxidase activity, was observed in tissues from vehicle- or inosine-treated rats (Y.G.C. and R.M.A., unpublished observations), suggesting that the observed functional improvements in inosine-treated animals were independent of changes in inflammatory state of the tissues. The contribution of adenosine receptor-dependent signaling in the observed effects of inosine on detrusor overactivity is currently under investigation.

Based on its favorable safety profile, as well as its anti-oxidant, anti-inflammatory and neurotrophic activities, inosine has recently completed evaluation in Phase II clinical trials for multiple sclerosis and Parkinson’s disease [28]. In the case of Parkinson’s disease, inosine administered orally was demonstrated to be safe and well tolerated by patients, with fewer adverse events in those receiving inosine versus placebo. Our demonstration of significant functional improvements in detrusor overactivity not only in a prevention study but also under conditions of existing neuronal damage suggests a novel use for inosine in treatment of neurogenic detrusor overactivity.
